# Dogs showed lower parasympathetic activity during mutual gazing while owners did not

**DOI:** 10.1186/s12576-023-00863-7

**Published:** 2023-05-15

**Authors:** Miho Nagasawa, Maaya Saito, Haruka Hirasawa, Kazutaka Mogi, Takefumi Kikusui

**Affiliations:** Department of Animal Science and Biotechnology, Azabu University, 1-17-71 Fuchinobe, Chuo-Ku, Sagamihara, Kanagawa 252-5201 Japan

**Keywords:** Attachment, Dog, Gaze, Heart rate variability, Strange Situation Test

## Abstract

The affiliative relationship between humans and dogs is compared to a mother–infant attachment relationship. We hypothesized that dog’s attachment behavior in negative emotional state aroused the owner’s attention toward the dog, that is, reduced parasympathetic activity. We measured heart rate variability in both dogs and humans during the Strange Situation Test to examine whether the owners' parasympathetic activity was decreased by being gazed at by their dogs. Our results in a short-term of 6 s before and after the moment the dog gazed at the human face indicated that dogs’ parasympathetic activity was lower when the dogs were gazing at their owners than when it was gazing at unfamiliar persons. Dog’s autonomic activity was also lower when the dogs were living with their owners for a longer period. However, we could not determine whether gaze from the dog affected the autonomic activity in humans as attachment behavior.

**Supplementary Information:**

The online version contains supplementary material available at 10.1186/s12576-023-00863-7.

## Background

In mammals, immature animals express behaviors to gain proximity and contact with their caregivers, such as mothers, and in response, caregivers exhibit protective behaviors. This behavior in immature animals is called attachment behavior. Attachment is a close emotional bond that is built with a specific partner and is found between mothers and offspring in mammals; however, the essence of attachment is a biobehavioral safety-regulating system that reduces negative emotions in immature animals [1]. In humans, it is thought that infants adjust their attachment behavior to the quantity and quality of their caregivers' nurturing behavior and adapt to the developmental environment surrounding them. The infant's belief that the caregiver, as an attachment target, provides a feeling of security when he/she needs protection can be of great help in the infant's establishment of the next relationship with others, and can be extremely important for the rest of his/her life [2, 3]. The Strange Situation Test (SST) was developed to understand individual differences in infant attachment and assess whether caregivers are secure bases for infants [4]. This test measures how infants direct their attachment behavior toward an attachment target, and how they use that target as a secure base under relatively stressful conditions. Infants are led into a novel experimental room and subjected to the mild stress of being confronted by an unfamiliar person and separated from their caregivers. If they regard their caregivers as a stable secure base, they will exhibit exploratory and playful behaviors when the caregivers are with them, while they will seem anxious when the caregivers are absent. However, whether the affiliative relationship formed between owners and dogs can be compared to a mother–infant-like attachment relationship is under debate [5]. The SST has been applied to assess such a human–dog relationship. The results suggest that the owner is a secure base for the dog and that the owner forms a bond with the dog as a specific individual [6–8].

There are differences between species in the signals that enable them to obtain care-giving that buffers the negative status, the so-called social buffering effects from others, including in attachment behavior [9]. For example, presenting the mother mouse with calls (pup ultrasonic vocalizations) and smells of her offspring elicits retrieval behavior [10]. In isolated marmosets, the voice of the bond-formed mating partner attenuated cortisol levels [11], and visual signals have a social buffering effect in sheep and humans [12, 13]. In human infants, gazing elicits a response from the caregiver, as does crying or smiling [14, 15]. Humans can use gazing behavior not only to obtain a simple visual information, but also as an output device for intentions and emotions in communication [16–18]. In general, direct gaze is said to indicate threat in wild animals [19, 20], but in canids, the more a species acts in groups, the more coloration around the eyes is emphasized, suggesting that they use their eyes as an output device for communication signals [21]. It has been suggested that dogs, which are said to have acquired human-like social cognitive abilities through convergent evolution with humans, use alternating gazes when faced with a task that is difficult to solve on their own or in response to the state of human knowledge [22]. These findings suggest that dogs use their own gaze as a communication signal to humans. In addition, urinary oxytocin levels of the owner increase when the dog gazes at the owner [23, 24], and intranasal administration of oxytocin to the dog increases gazing behavior toward the owner [24]. As oxytocin has been shown to promote attachment behavior and the caregiver's response to it, the dog’s gaze may function as an attachment behavior, eliciting nursing behavior from humans despite their differences in species. Experiments with oxytocin administration in Japanese dogs showed that the sympathetic nervous system was activated in the owner when gazed at by the dog after oxytocin administration to the dog [25]. If attachment is a biobehavioral safety-regulating system that reduces negative emotions in immature animals through proximity and contact with caregivers, it is expected that caregivers, who are recipients of the attachment behavior, will also recognize that the sender is in a negative situation and will increase their level of arousal. Therefore, in the study of Japanese dogs, the increase in sympathetic activity in owners who were gazed at by their dogs can be interpreted as being due to the attention aroused by the attachment behavior of the dogs.

The heart rate is controlled by both the sympathetic and parasympathetic nerves, and the balance between these two systems determines the R–R interval (RRI), which is thought to include fluctuations and is unstable. Therefore, heart rate variability (HRV) is a useful index for measuring autonomic nervous system activity and varies with emotional state [26, 27]. The root mean square of the successive differences in RRI (rMSSD) reflects the beat-to-beat variance in heart rate and is the primary value for estimating vagally mediated changes reflected in HRV. In contrast, both the sympathetic and parasympathetic nervous systems contribute to the mean of the standard deviations of the RRI (SDNN). Thus, HRV parameters are useful indicators for measuring autonomic nervous system activity attributable to emotional states [26, 27]. In animals, several studies have been conducted on the relationship between HRV and negative emotional state from an animal welfare perspective [28–33]. In addition to stress, recent studies have shown a relationship between positive emotions and HRV, with negative emotions associated with decreased rMSSD, and positive emotions with decreased SDNN [34, 35].

Based on the above, we hypothesized that when dogs show attachment behavior in negative emotional state, the owners’ attentions are aroused toward the dogs, that is, reduced parasympathetic activity. To clarify whether the gazing behavior of dogs functions as an attachment behavior toward humans, we measured the HRV in both dogs and humans during SST, which is assumed to evoke the attachment behavior in dogs. We also investigated whether dogs exhibit gazing behavior toward specific persons (owner), who are the targets of their attachments, in the SST as compared to an unfamiliar person and whether the gazing behavior of dogs raises the arousal level and decreases parasympathetic activity in owners. We finally examined whether the gazing behavior of dogs synchronizes the emotional state between the owner and the dog, which should result from a reduction in parasympathetic activity in both dogs and owners.

## Methods

### Subjects

A total of 22 pairs of pet dogs (from 10 months to 12 years old, mean ± SD = 5.83 ± 3.4 years; 11 female dogs and 11 male dogs, 4 dogs were unneutered, Table 1) and their owners (18 females and 4 males) participated. They were recruited from animal hospitals, parks, and our university, and we ensured that both the dogs and their owners were in good health. Eleven female university students whom the subject dogs met for the first time participated as the “stranger” in the SST. Some students participated in the experiment more than once. All experimental procedures were approved by the Animal Ethics Committee of Azabu University (#180410-1) and the Ethical Committee for Medical and Health Research Involving Human Subjects of Azabu University (#052). The consent of the owners and participants was obtained after explaining the experimental procedures and the owners could stop participation at any time for any reason.

**Table 1:** Information of subject dogs

ID	Breed	Owning period (year)	Sex
1	Labrador retriever	1.33	Female
2	Goldendoodle	1.48	Male
3	Poodle (toy)	2.43	Male
4	Jack Russell Terrier	4.55	Male
5	Poodle (toy)	10.49	Male
6	MIX	0.25	Female
7	Japanese Terrier	4.78	Male (intact)
8	MIX	12.00	Female
9	Whippet	4.64	Female
10	Shih Tzu	7.47	Female
11	Border collie	5.95	Female
12	MIX	5.77	Female (intact)
13	Bernese Mountain Dog	6.43	Female
14	Poodle (toy)	1.96	Male (intact)
15	Saluki	2.04	Female
16	Poodle (toy)	9.13	Male (intact)
17	Border collie	3.18	Male
18	Dalmatian	12.49	Female
19	MIX	0.33	Female
20	Shiba Inu	2.49	Male
21	Dalmatian	11.12	Male
22	Golden retriever	4.24	Male

F: female dog, M: male dog

### Apparatus

The experiment was conducted in an experimental room (4 × 6 m) at Azabu University, which was the first visit for both dogs and their owners. Two chairs were placed at least 1 m apart in the center of the experimental room, with the dog toys (ball, stuffed animal, and rope) between the two chairs. Digital video cameras were used to record the behavior, with one placed on the ceiling and two at locations where they could capture the entire room.

### Procedure

**Fig. 1:**
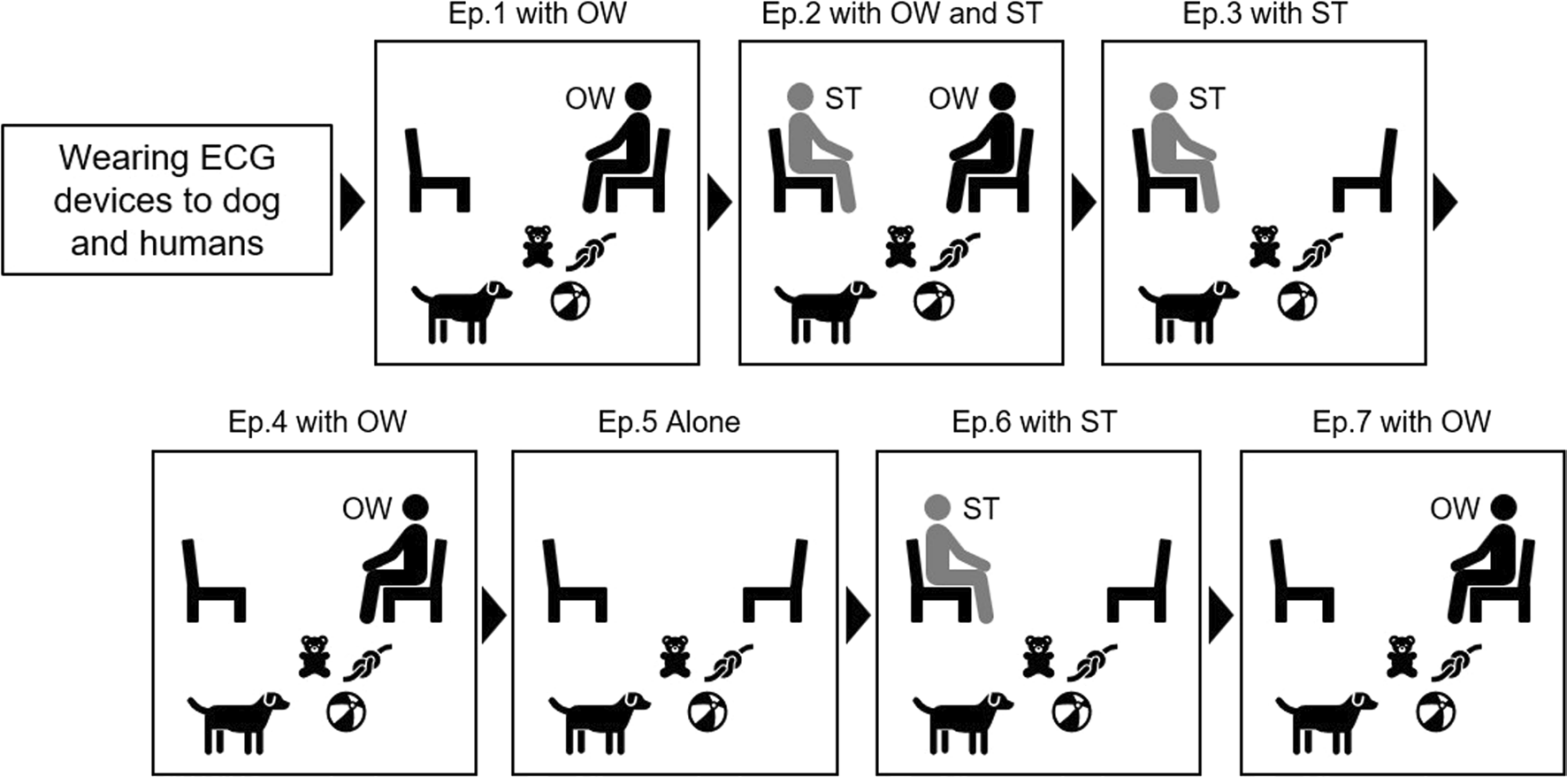
Procedure of experiment. The SST consists of episodes (Ep.) in which the dog is with its owner (OW), with a stranger (ST), with the owners and stranger, and alone in the experimental room. The type of behavior a dog exhibits toward its owner measures whether it utilizes its owner as a safety base. This procedure is a modified version of Gásci et al. [36]. Each episode lasted approximately three minutes

A schematic of the procedure is illustrated in Fig. 1. The SST in this study was conducted using the method of Gácsi et al. [36]. While Gácsi et al.'s method was performed for approximately 2 min per episode, to collect the number of data needed for the analysis of HRV parameters, each episode took approximately 3 min, and the experiment lasted a total of 21–25 min in this study. After informing owners of the experimental contents, the owners who signed the informed consent participated in the experiment. The owners were asked to complete the attribute data, such as the age of dogs, sex, neutering status, and duration of ownership. The dog, its owner, and the student in the role of a stranger were equipped with electrocardiogram (ECG) devices before entering the experimental room. Compact multifunction sensors (Faros 360°; Bittium, Kuopio, Finland) were used as the ECG recording devices. After a 5-min habituation to the ECG devices, the dog and owner entered the experimental room and participated in the SST. The SST consisted of seven episodes: three episodes of the dog and its owner, one episode of the dog, its owner, and a stranger, two episodes of the dog and a stranger, and one episode of the dog alone (details are shown in Fig. 1 and Additional file 1: Table S1). When giving instructions to the owners during the experiment, a knock was used as a cue, or the owner was guided by an earpiece transceiver. The behavior of the dogs during the SST was recorded by three video cameras and was analyzed after the experiment was completed.

### Analysis

#### Behavioral analysis

The behavior of dogs during SST was recorded using video cameras (GoPro HERO3; GoPro, Inc., USA), and the duration of dog behavior was coded after the experiment using a free event-logging-software (BORIS) [37]. In addition to exploration, play, and physical contact with humans which were analyzed in the previous studies [6, 36], gazing behavior towards humans was coded in this analysis. The criteria for exploration, play, and physical contact followed Gasci et al. [36], and gazing behavior followed Nagasawa et al. [24]. These behaviors were coded by two experimenters, and we confirmed that Cohen's Kappa of their coding exceeded 0.9 for all behaviors. Excluding the time the dog was out of frame, such as crawling under a chair, the proportion of time the behavior was observed during each episode was used in the analysis.

#### ECG recordings of the dogs and their owners and analysis

ECG measurements were performed as described previously [35]. ECG induction in each dog was performed using an M-X lead. To record ECG in the dogs without shaving, we created a band that combined bandaging tape (3 M Vetrap bandaging tape; 3 M, Tokyo, Japan) and three disposable electrodes (monitoring electrode 2228; 3 M, Tokyo, Japan). We parted the hair on each dog such that the skin was visually observable in the manubrium and xiphisternum. Ultrasound gel (Aquasonic Clear; Parker, New Jersey, USA) was applied to the skin surface. We wrapped three electrodes and an ECG recording device directly onto the skin of each dog using an elastic bandage. For the owner, ECG measurements were performed using a CC5 lead. The owner was asked to fix the three electrodes along the fifth costa. The ECG sampling rate was set to 1000 Hz.

The RRI detection from the ECG analysis was the same as that used in our previous report [35]. Briefly, we detected R waves using the original MATLAB script from the recorded ECG data together with visual confirmation and then calculated the RRI. Subsequently, HRV parameters were calculated using the following setting: the length of the analysis time window was set to 15 s, and the time windows were staggered by 5 s and overlapped by 10 s each. The HRV parameters were calculated for each time bin: SDNN, which is the index of the autonomic nervous systems, rMSSD, which is the index of the parasympathetic nervous system, and mean R–R intervals (mean RRI). If the RRI values were not detected owing to mechanical errors for more than 10% of occurrences in each time bin, the time bin data were excluded from the analysis. Thirteen strangers with incomplete data were excluded from further analysis because of serious ECG artifacts due to defective electrode contacts during the SST episodes. We calculated the correlation coefficients of each HRV for each episode to examine emotional synchronization between humans and dogs for Analysis 2. The median of each HRV per episode was also calculated and used in Analyses 1 and 2. In Analysis 3, after extracting the dog's gaze behavior toward the human participant when it was at least 6 s, ECG data from 6 s before and after the moment the dog gazed at the human face were clipped and averaged every 2 s for analysis.

#### Statistical analysis

We used the data obtained to perform the following analyses: Analysis 1: whether dogs gaze at their owners rather than at strangers in the SST, and whether HRV parameters of the dogs can be used to explain the duration of their gazing at humans; Analysis 2: whether the duration of a dog’s gazing behavior can explain human HRV parameters and whether gazing duration of the dogs can explain the emotional synchronization between humans and dogs; and Analysis 3: whether HRV parameters change before and after the dog gazes at the human. Previous studies have pointed out the influence of repetition and order of episodes [7, 38]. Episodes 4 and 7, in which the dog was with the owner (OW episodes), and episodes 3 and 6, in which the dog was with a stranger (ST episodes), were selected and used in the analysis. The dog's behavior was compared between each episode, and no statistical difference was found in behavior due to the order of these episodes (Additional file 1: Figure S1). For comparisons between OW and ST episodes, a Wilcoxon signed-rank test was conducted on dog behaviors and dog and human HRV parameters. To determine whether dogs’ gaze toward humans is involved in changes in the autonomic activity of dogs and humans, we used a linear mixed model (LMM) with three HRV parameters, dog behavior, the duration of dog ownership, dog sex, and OW or ST episodes. We analyzed these data using a statistical software (JMP ver.14.2.0, JMP Japan).

## Results

### Analysis 1: behaviors and HRVs in dogs

**Fig. 2:**
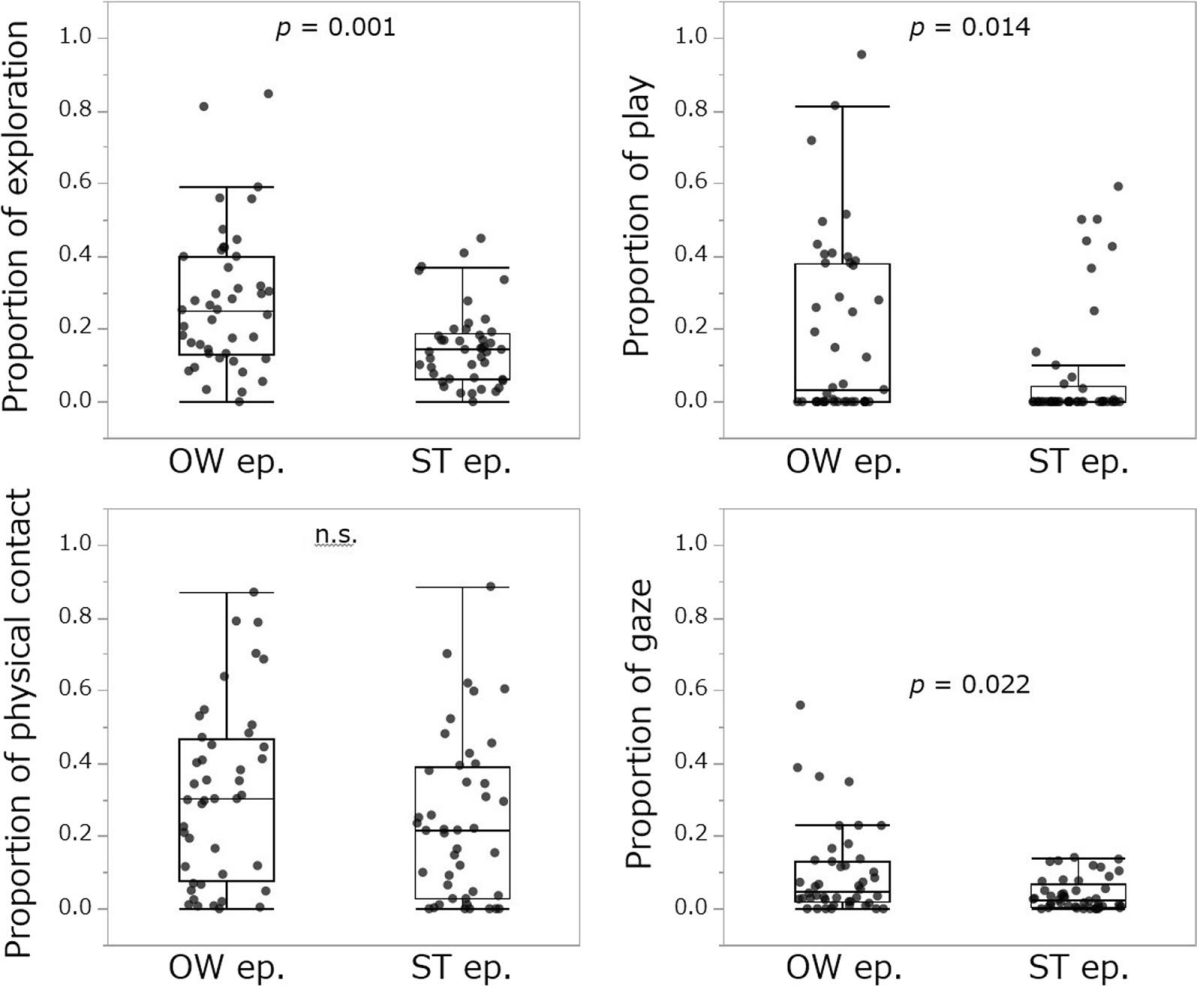
Comparisons of behavior in dogs between OW and ST episodes during Strange Situation Test. OW ep. indicates the episodes in which only the owner (OW episodes) and dog participated and ST ep. indicates the episodes in which only the stranger and dog participated (ST episodes). The vertical axis indicates the proportion of the behavior expressed in a single episode (approximately 3 min). Dots indicate individual data

**Fig. 3:**
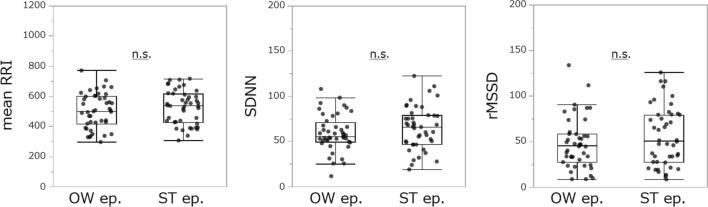
Comparisons of HRV parameters in dogs between OW and ST episodes during Strange Situation Test. OW ep. and ST ep. indicate episodes in which only the owner and the dog participated (OW episodes) and episodes in which only the stranger and the dog participated (ST episodes), respectively. Dots indicate individual data

Four behaviors of dogs during SST were compared between the OW and ST episodes. The durations of exploration (median of proportion: OW episode = 0.256, ST episode: 0.144, *z* = − 3.380, *p* = 0.001), play (OW episode = 0.085, ST episode = 0.000, *z* = − 2.463, *p* = 0.014) and gaze (OW episode = 0.042, ST episode = 0.026, *z* = − 2.285, *p* = 0.022) were significantly longer in the OW episodes than the ST episodes. No significant differences were found in physical contact (OW episode = 0.297, ST episode = 0.216, *z* = − 1.067, *p* = 0.108) (Fig. 2). Comparisons of HRV between OW and ST episodes in dogs showed no significant differences in HRV parameters (Fig. 3). Whether the gaze duration increases with the emotional state of the dog was examined in an LMM using dog sex, ownership duration, episodes (OW ep. or ST ep.), each HRV index, and the interaction between episodes and each HRV parameter as explanatory variables, and the dog ID as a random effect. However, none of the HRVs explained the duration of the dog's gaze behavior. When any of the HRV parameters were included in the explanatory variables, dogs gazed at their owners longer than strangers (Table 2).

**Table 2:** Results of LMM of the relationship between each dog’s HRV parameter and gazing duration of the dog

		*β*	*t*	*p*	Adjusted *R*^*2*^
Mean RRI	Intercept		0.698	0.487	0.243
	Dog's sex (female dog)	0.107	0.935	0.353	
	Owning period	0.031	1.980	0.051	
	Episodes (OW ep.)	***0.166***	***2.990***	***0.004***	
	meanRRI	− 0.210	− 0.822	0.414	
	Episodes (OW ep.) * meanRRI	0.182	0.745	0.458	
SDNN	Intercept		− 0.064	0.949	0.235
	Dog's sex (female dog)	0.075	0.657	0.513	
	Owning period	0.030	1.907	0.060	
	Episodes (OW ep.)	***0.171***	***3.062***	***0.003***	
	SDNN	0.000	− 0.002	0.999	
	Episodes (OW ep.) * SDNN	− 0.075	− 0.553	0.582	
rMSSD	Intercept		0.016	0.988	0.232
	Dog's sex (female dog)	0.074	0.651	0.517	
	Owning period	0.031	1.956	0.054	
	Episodes (OW ep.)	***0.170***	***3.040***	***0.003***	
	rMSSD	− 0.013	− 0.148	0.883	
	Episodes (OW ep.) * rMSSD	− 0.006	− 0.076	0.940	

We examined whether dog HRV parameters influence the duration of dog's gazing behavior toward human participants

*β* indicates partial regression coefficient

Dog’s sex: female dog against male dog

Episodes: OW episodes (dogs with owners) against ST episodes (dogs with strangers)

Bold italicized numbers indicate that the results were statistically significant

### Analysis 2: HRVs in humans

**Fig. 4:**
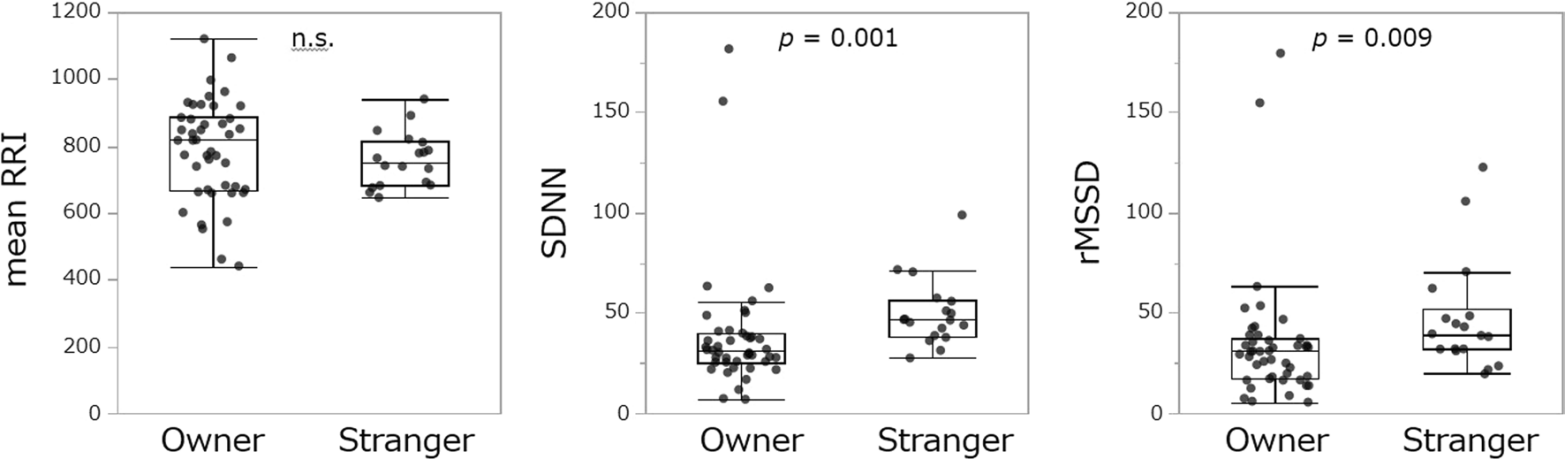
Comparisons of HRV parameters between owners and strangers during Strange Situation Test. Dots indicate individual data

Each parameter of human HRV was compared between owners (in OW episodes) and strangers (in ST episodes). SDNN (*z* = 3.500, *p* = 0.001) and rMSSD (*z* = 2.613, *p* = 0.009) were lower in owners than in strangers (Fig. 4). To examine the influence of dog behavior on human HRVs, LMM was conducted with each HRV as the objective variable, using dog’s sex, ownership duration, human participant (owner or stranger), and the interaction between dog behaviors and human participant (owner or stranger) as explanatory variables. We found that rMSSD (*t* = − 2.113, *p* = 0.041) was lower in owners than in strangers (Table 3). However, we did not find any significant effect of dog behavior on human HRVs.

**Table 3:** Results of LMM of the relationship between dog behaviors and each HRV parameter in human participants

	MeanRRI			SDNN			rMSSD		
	*β*	*t*	*p*	*β*	*t*	*p*	*β*	*t*	*p*
Intercept		1.068	0.290		− 1.746	0.087		− 0.845	0.430
Dog's sex (female dog)	− 0.057	− 0.515	0.609	0.143	1.506	0.138	− 0.082	− 0.588	0.559
Owning period	− 0.016	− 0.976	0.334	0.013	0.937	0.353	− 0.011	− 0.550	0.585
Episodes (OW ep.)	***0.254***	***4.098***	** < *0.001***	0.046	0.874	0.386	0.035	0.387	0.701
Episodes (OW ep.) * Exploration	0.042	0.411	0.683	0.086	0.996	0.324	− 0.042	− 0.352	0.726
Episodes (OW ep.) * Play	0.037	0.552	0.583	0.039	0.676	0.502	0.079	1.032	0.307
Episodes (OW ep.) * Physical contact	− 0.054	− 0.631	0.531	− 0.072	− 0.984	0.330	0.094	0.839	0.406
Episodes (OW ep.) * Gaze	0.182	1.776	0.082	− 0.009	− 0.108	0.915	0.198	1.682	0.099
Adjusted *R*^*2*^	0.166			0.002			0.041		

*β* indicates partial regression coefficient

Dog’s sex: female dog against male dog

Episodes: OW episodes (dogs with owners) against ST episodes (dogs with strangers)

Bold italicized numbers indicate that the results were statistically significant

We also analyzed the influence of dog behavior on the correlation between dog and human participant’s HRVs by LMM using the same explanatory variables. As the results showed, the correlation coefficient of mean RRI was higher in OW episodes (*t* = 4.098, *p* < 0.001) (Table 4). However, we did not find any significant effect of dog behavior on the correlation coefficient HRVs.

**Table 4:** Results of LMM of the relationship between dog behaviors and the correlation coefficient between dog and human participant’s HRVs

	MeanRRI			SDNN			rMSSD		
	*β*	*t*	*p*	*β*	*t*	*p*	*β*	*t*	*p*
Intercept		17.201	< 0.001		2.160	0.326		1.517	0.179
Dog's sex (female dog)	− 0.056	− 1.160	0.251	− 0.006	− 0.036	0.972	− 0.068	− 0.371	0.713
Owning period	0.000	− 0.063	0.950	− 0.022	− 0.932	0.356	− 0.036	− 1.318	0.193
Human participant (owner)	− 0.011	− 0.365	0.717	− 0.218	− 1.922	0.069	*** − 0.262***	*** − 2.113***	***0.041***
Human participant (owner) * Exploration	0.071	1.676	0.100	0.038	0.278	0.782	0.192	1.202	0.235
Human participant (owner) * Play	0.042	1.555	0.126	0.051	0.601	0.551	0.058	0.570	0.571
Human participant (owner) * Physical contact	0.037	0.984	0.329	− 0.006	− 0.044	0.965	− 0.073	− 0.502	0.618
Human participant (owner) * Gaze	0.034	0.798	0.428	− 0.013	− 0.096	0.924	0.009	0.054	0.957
Adjusted *R*^*2*^	0.123			0.047			0.200		

*β* indicates partial regression coefficient

Dog’s sex: female dog against male dog

Human participants: owner against stranger

Bold italicized numbers indicate that the results were statistically significant

### Analysis 3: changes in HRV owing to gaze in dogs and human participants

Finally, we clipped the HRV data for 12 s (6 s before and 6 s after the moment dogs gazed at human participants) to determine whether human participant’s and dog’s HRVs changed before and after the moment of dog's gaze. We obtained 19 data points from OW episodes of 13 pairs and 19 data points from ST episodes of 15 pairs. LMM was conducted using dog’s sex, ownership duration, time (total 12 s before and after the moment dogs gazed at human participants), and episodes (OW ep. Or ST ep.); however, no changes in HRV were found in both dogs and human participants during the 12 s. In dogs, the mean RRI (*t* = − 2.407, *p* = 0.016), SDNN (*t* = − 5.688, *p* < 0.001), and rMSSD (*t* = − 4.815, *p* < 0.001) were significantly lower when they gazed at their owners than when they gazed at strangers. Dogs that had been owned for long periods also showed a low SDNN (*t* = − 2.502, *p* = 0.024). In human participants, SDNN (*t* = − 20.445, *p* < 0.001) and rMSSD (*t* = − 21.801, *p* < 0.001) were significantly lower in owners than in strangers (Table 5).

**Table 5:** Results of LMM of the change in each HRV parameter for 6 s before and 6 s after the moment dogs gazed at human participants

	meanRRI			SDNN			rMSSD		
	*β*	*t*	*p*	*β*	*t*	*p*	*β*	*t*	*p*
*Dog's HRV*									
Intercept		60.545	< 0.001		22.740	< .0001		14.246	< .0001
Dog's sex (female dog)	− 0.079	1.231	0.242	0.030	− 0.249	0.808	0.080	− 0.440	0.668
Owning period	0.015	0.872	0.393	*** − 0.087***	*** − 2.502***	***0.024***	− 0.098	− 1.897	0.075
Episodes (OW ep.)	*** − 0.015***	*** − 2.407***	***0.016***	*** − 0.097***	*** − 5.688***	** < *0.001***	*** − 0.105***	*** − 4.815***	** < *0.001***
Time	0.000	− 0.300	0.764	− 0.002	− 0.473	0.636	0.003	0.523	0.601
Episodes (OW ep.) * Time	− 0.001	− 0.663	0.508	0.005	1.254	0.210	0.001	0.278	0.781
Adjusted *R*^*2*^	0.622			0.586			0.658		
*Human HRV*									
Intercept		***97.017***	** < *.0001***		***14.679***	** < *.0001***		***10.641***	** < *.0001***
Dog's sex (female dog)	0.041	− 1.085	0.298	0.091	− 0.673	0.513	0.170	− 0.961	0.354
Owning period	0.000	− 0.008	0.994	− 0.034	− 0.793	0.442	− 0.031	− 0.557	0.587
Human participant (owner)	− 0.009	− 1.950	0.052	− 0.404	*** − 20.440***	** < *.0001***	*** − 0.413***	*** − 21.800***	** < *.0001***
Time	0.001	0.969	0.333	− 0.001	− 0.358	0.721	− 0.002	− 0.442	0.659
Human participant (owner) * Time	0.000	0.248	0.805	− 0.002	− 0.379	0.705	0.001	0.348	0.728
Adjusted *R*^*2*^	0.605			0.564			0.634		

*β* indicates partial regression coefficient

Dog’s sex: female dog against male dog

Episodes: OW episodes (dogs with owners) against ST episodes (dogs with strangers)

Human participants: owner against stranger

Bold italicized numbers indicate that the results were statistically significant

## Discussion

The results showed that dogs gazed at owners, as well as explored and played, more than with strangers during the SST. Exploration and play are behaviors that human infants exhibit when they regard their caregivers as a secure base; this applies to the relationship between dogs and their owners. Therefore, in this study, the fact that dogs exhibited many of these behaviors during episodes with their owners indicates that as in previous studies [6, 36], they distinguished between owners and strangers and expressed attachment-related behaviors. In addition, SST was developed to elicit human infants' attachment behavior to their caregivers by arousing their anxiety about visiting a place for the first time. In the previous study, dogs gazed at their owners rather than at strangers in an experimental setting in which dogs feel anxious [23, 24], and such dog’s gazing increased urinary oxytocin levels in the owner, which in turn increases the nursing behavior of the owner toward the dog [23, 24]. In the current study, in SST, a setting that arouses anxiety in dogs, there was more frequent gazing toward the owner than toward the stranger. Thus, the dogs may have been showing gazing behavior to their owners in a situation like this as an attachment behavior. In an analysis using HRV, the median value for each episode, none of the HRV parameters of the dogs showed significant differences between owners and strangers, that is, differences in emotional state. We also examined whether the HRV of a dog was related to the duration of gazing behavior and found that none of the HRV parameters could explain gazing behavior. The first reason for these results is that the median HRV of the 3-min episodes was used in the analysis, which may have erased the minute changes in autonomic activity during each episode. Second, it is possible that different types of gazing behavior, which may have been an attachment behavior or may have been just looking, were mixed during each episode. The former is discussed later, and analyzed in detail in Analysis 3.

Next, we analyzed changes in the HRV of owners and strangers who were recipients of attachment behavior. First, we compared the HRV parameters of the owners and strangers during the episodes and found that both the SDNN and rMSSD of the owners were lower than those of the strangers, suggesting a decrease in parasympathetic activity in the owners [39]. In general, reduced HRV at rest is associated with stress, anxiety, worry, or panic [40]. Although the human participants in this experiment were not at full rest, there was no significant difference in the mean RRI between owners and strangers, suggesting that this decrease in parasympathetic activity was not because of higher physical activity than that of strangers but rather to stress. However, most dogs in this experiment were tamed and non-aggressive, and the experimenters playing the role of a stranger were familiar with the experimenters, the procedure, and the experimental room, so it may not have been a tense situation during the SST, which may have also contributed to the higher parasympathetic activity than that of the owners. Second, gazing duration of dogs was not related to any HRV parameters in human participants. Because a previous study has shown that dog and owner emotions synchronize when owners are subjected to mental stress [41], we also investigated factors affecting the correlation between HRV parameters in human participants and dogs. We found that the correlation between dog and human participant in mean RRI was higher during OW episodes than ST episodes. This result indicates that physical activity may be more synchronized between the dog and its owner than the dog and stranger.

Finally, we examined whether gazing by dogs caused changes in autonomic activity in both dogs and human participants; however, neither dogs nor human participants showed any changes between before and after the moment dogs gazed at human participants. However, in dogs, although there was no difference in the median per episode of HRV between OW and ST episodes in analysis 1, analysis using data for short periods of time before and after the dog gazed at its owner showed that parasympathetic activity was lower when the dog was gazing at the owner than the stranger. The dog’s mean RRI also decreased, suggesting a possible influence of physical activity [42]. However, dog’s SDNN decreased during OW episodes compared to ST episodes, that is, autonomic activity is decreased, suggesting that the assumed influence of physical activity can be ruled out. The SDNN was significantly lower in the dogs that lived with their owners longer. In a previous study, only SDNN was low in the positive emotional condition in which the dogs were gently touched by their owners [35]. However, as described above, this analysis shows that dog's SDNN and rMSSD were significantly lower in OW episodes than ST episodes, therefore, rMSSD also tended to be low in this study, this a decrease in SDNN may be associated with decreased parasympathetic activity [29]. Therefore, the dog may have been in a negative emotional state when gazing at its owner, and the dog may have acquired this use of gazing behavior toward humans in their daily lives with humans. At the very least, for dogs, gazing at their owners may be an emotional signal in their interactions with their owners. Owners, in contrast, showed a reduction in autonomic and parasympathetic activity when gazed at by their dogs as compared to strangers, as well as analysis 1. It is possible that the SST experimental procedure was also generally tense for the owners regardless of dog's gazing behavior because the owners had to follow instructions from the experimenter in a novel place for them, whereas the specific experimenter repeatedly played the role of the "stranger" and was familiar with this procedure. In addition, only pairs for which 12 s of data could be clipped were used in the analysis; therefore, dogs that frequently gazed at humans for short periods of time were excluded. Therefore, it is possible that not all gazing behaviors, such as attachment behaviors, were included in the analysis.

## Conclusions

In summary, dogs were found to gaze at their owners more often than at unfamiliar persons during SST. In addition, a short-term analysis, before and after the moment of dog's gazing at the human participant, suggested that parasympathetic activity was lower when the dog was gazing at its owner than when it was gazing at an unfamiliar person, and even lower when the dog was living with its owner for a longer period. Therefore, we found the possibility that there is an association between dog’s negative emotional state and gazing behavior, and dogs learned how to use gazing behavior toward humans in their lives with humans. While we could not find any subtle changes in the owners owing to eye contact with the dog, and gazing behavior from dogs also did not cause emotional synchronization between dogs and their owners. Therefore, we could not determine whether gaze from the dog affected the autonomic activity in humans as attachment behavior in this study. A limitation of this study is that we did not restrict physical activity in both human participants and dogs; therefore, we could only infer from the mean RRI whether the autonomic activity was caused by exercise or stress. Furthermore, because many of the heart rate data from strangers had missing values and could not be used in the analysis, a more reliable method for attaching the ECG device needs to be investigated. Four of the 22 owners in this study were male. Although it is conceivable that human sex differences may well influence dog behavior [43], the number of male owners was small in this study, so the analysis was conducted without considering gender differences. Future experiments should take more detailed attributes into account.

### Supplementary Information

**Additional file 1:** The procedure of Strange Situation Test (Table S1) and Behavior in dogs between OW and ST episodes during Strange Situation Test (Figure S1).

## Data Availability

All data sets are shared by the corresponding author upon the request.
