# Supplementary material for: Dogs showed lower parasympathetic activity during mutual gazing while owners did not
Source: J Physiol Sci. 2023 May 15;73:9. doi: 10.1186/s12576-023-00863-7 (PMC10715594; doi:10.1186/s12576-023-00863-7)
Supplement: Supplementary file 1 — Additional file 1: The procedure of Strange Situation Test (Table S1) and Behavior in dogs between OW and ST episodes during Strange Situation Test (Figure S1). [file 12576_2023_863_MOESM1_ESM.pdf]

Table S1. The procedure of Strange Situation Test.

Episode 1	The owner (OW) enters the laboratory with the dog, sits down in a chair, and reads the instructions for the experimental procedure. After 90 sec, the OW either plays with or pets the dog, depending on its behavior. When the Stranger (ST) enters the room, the OW stops interacting with the dog and returns to the chair.
Episode 2	After ST enters the room, she stops for 5 sec to allow the dog to respond, and then sits in a chair. Forty sec later, OW and ST begin a conversation, which lasts 45 sec, after which ST either plays with or pets the dog, depending on the dog's behavior. Ow quietly leaves the room.
Episode 3	After 90 sec, ST stops interacting with the dog and sits in the chair. If the dog is nearby, ST strokes the dog.
Episode 4	The OW enters the room after calling the dog's name at the door, stands still for 5 seconds, and then sits down. The OW either plays with or pets the dog, depending on its behavior. 90 sec later, the OW stops interacting with the dog and sits down in the chair. If the dog is nearby, the OW pet it. At the end of the episode, the OW says to the dog, "Wait here," and then leaves the room.
Episode 5	The dog is closed in the room for 3 min.
Episode 6	After entering the room, ST stops for 5 sec and then plays or pets, depending on its behavior. After 90 sec, ST stops interacting with the dog and sits down in the chair. If the dog is nearby, ST pet it. When OW enters the room, ST stops interacting with the dog.
Episode 7	The OW enters the room after calling the dog's name at the door, stands still for 5 seconds, and then sits down. Meanwhile, ST leaves the room. The OW either plays with or pets the dog, depending on its behavior. 90 sec later, the OW stops interacting with the dog and sits down in the chair. If the dog is nearby, the OW pet it. The experiment ends when the experimenter enters the room.

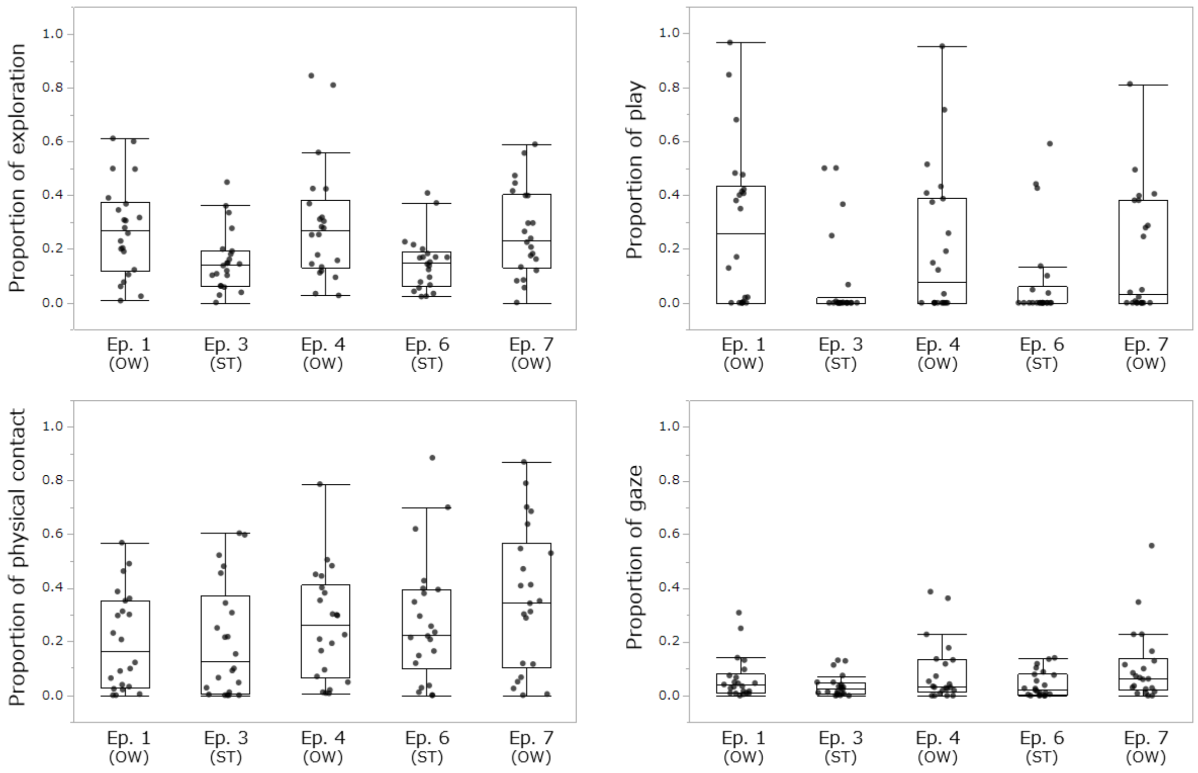

Figure S1. Behavior in dogs between OW and ST episodes during Strange Situation Test.
OW ep. indicates the episodes in which only the owner (OW episodes) and dog participated and ST ep. indicates the episodes in which only the stranger and dog participated (ST episodes). The vertical axis indicates the proportion of the behavior expressed in a single episode (approximately 3 minutes). Dots indicate individual data. We excluded ep. 2, in which both owner and stranger participated, and ep. 5, in which only dog remained. Ep. 4 and 7 as OW episodes and Ep. 3 and 6 as ST episodes were used in the analysis. No significant differences were found between each episode (ep. 4 vs. 7, 3 vs. 6).
